# Attenuation of Stimulated Accumbal Dopamine Release by NMDA Is Mediated through Metabotropic Glutamate Receptors

**DOI:** 10.1021/acschemneuro.2c00777

**Published:** 2023-04-06

**Authors:** Felicity S. E. Spencer, Maria Glodkowska, Anna I. Sebold, Ersin Yavas, Andrew M. J. Young

**Affiliations:** † School of Sport, Exercise and Rehabilitation Sciences, University of Birmingham, Edgbaston, Birmingham B15 2TT, U.K.; ‡ Department of Psychology, Bartın University, Bartın 74100, Turkey; § School of Psychology and Vision Sciences, University of Leicester, Lancaster Road, Leicester LE1 9HN, U.K.

**Keywords:** Brain slices, dopamine, fast-scan cyclic voltammetry, metabotropic glutamate receptors, *N*-methyl-d-aspartate (NMDA), nucleus accumbens

## Abstract

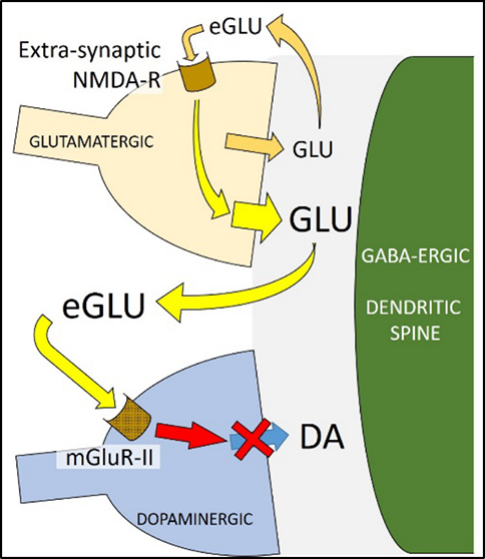

Electrically stimulated dopamine release from the nucleus accumbens is attenuated following application of *N*-methyl-d-aspartate (NMDA), which is likely to be mediated indirectly through intermediary neuronal mechanisms rather than by a direct action on dopamine terminals. On the basis of known modulatory processes in nucleus accumbens, the current experiments sought to test whether the effect of NMDA was mediated through cholinergic, GABA-ergic, or metabotropic glutamatergic intermediate mechanisms. Fast-scan cyclic voltammetry was used to measure electrically stimulated dopamine release in nucleus accumbens of rat brain slices *in vitro.* Stimulated dopamine release was attenuated by NMDA, confirming previous findings, but this attenuation was unaffected by either cholinergic or GABA-ergic antagonists. However, it was completely abolished by the nonselective group I/II/III metabotropic glutamate receptor antagonist α-methyl-4-carboxyphenylglycine (MCPG) and by the selective group II antagonist LY 341396. Therefore, group II metabotropic glutamate receptors, but not acetylcholine or GABA receptors, mediate the attenuation of stimulated dopamine release caused by NMDA, probably by presynaptic inhibition through receptors located extra-synaptically on dopamine terminals. This provides a plausible mechanism for the documented role of metabotropic glutamate receptor systems in restoring deficits induced by NMDA receptor antagonists, modeling schizophrenia, underlining the potential for drugs affecting these receptors as therapeutic agents in treating schizophrenia.

## Introduction

The mesolimbic pathway projects from the ventral tegmental area (VTA) in the midbrain to the nucleus accumbens (NAc) in the forebrain. This pathway is primarily dopaminergic and is critically involved in controlling emotional responding and reward,^1,2^ with dopamine release at the terminals particularly important in the motivation and reinforcement of goal-directed behavior.^3^ Dysfunction in this pathway has been associated with schizophrenia^4−6^ probably through dysregulation of glutamate/dopamine interactions.^6^ In particular *N*-methyl-d-aspartate (NMDA) receptors are likely to be important, since noncompetitive NMDA receptor antagonists, ketamine and phencyclidine (PCP), (1) cause behavioral changes in normal people resembling symptoms of schizophrenia,^7,8^ (2) enhance symptom expression in schizophrenia sufferers,^8,9^ and (3) cause changes in behaviors in experimental animals which resemble changes seen in schizophrenia.^10,11^ For this reason, noncompetitive NMDA antagonist treatment is used as an animal model of schizophrenia.^12−14^

One important site of glutamate/dopamine interaction is at the terminals of mesolimbic dopamine neurones in NAc. Using fast-scan cyclic voltammetry (FSCV) in rat brain slices *in vitro,* Yavas and Young^14^ demonstrated that NMDA dose-dependently attenuated electrically stimulated dopamine release in the NAc. However, this finding is surprising for two key reasons. First, NMDA receptors are typically excitatory,^15^ and therefore it would be expected that activation of these receptors would increase dopamine release. Second, evidence suggests that NMDA receptors are not widely located on dopamine terminals in this region (refs (16) and (17) but see ref (18)). Therefore, it is likely that the effect of NMDA on stimulated dopamine release in NAc is mediated via an intermediary mechanism, probably involving cholinergic, GABA-ergic, and/or metabotropic glutamatergic (mGluR) receptor mediated processes.

Acetylcholine containing interneurons form only around 5% of the total number of neurones in the striatum, but their extensive arborization makes them exquisitely placed to provide powerful modulatory influence on dopamine release through local actions on the mesolimbic terminals in NAc.^19−22^ There are two classes of cholinergic receptor (AChR): nicotinic and muscarinic. The activation of nicotinic AChR by nicotine enhances dopamine release in the NAc, which is disrupted by subchronic PCP pretreatment.^23^ It is established that nicotinic AChRs are present on dopamine terminals in this region^24,25^ and that activation of these receptors leads to dopamine release here.^26,27^ Furthermore, activation of AChR with carbachol in freely moving rats enhances locomotor activity, a behavior primarily under the control of accumbal dopamine. Taken together, these studies suggest that nicotinic AChR modulates dopamine release in the NAc.

The role of muscarinic AChR on dopamine release from the NAc is complex. Activation of the M5 subgroup of muscarinic AChR enhances dopamine release in the midbrain, but M2 and M4 muscarinic AChRs inhibit dopamine neurones in the striatum.^28^ Furthermore, local application of muscarinic AChR antagonists into NAc disrupted reward-seeking,^29^ a behavior under the control of mesolimbic dopamine. This was confirmed by additional experimentation with FSCV suggesting further that muscarinic AChRs have some control over dopamine release here.^30^ It is therefore plausible that NMDA activates cholinergic interneurons, which in turn inhibit the release of dopamine from mesolimbic terminals in NAc.

GABA-ergic systems also have an important regulatory role in NAc, through both GABA-A and GABA-B receptors. Previous FSCV experiments in rat brain slices *in vitro*(14) demonstrated that simultaneous application of the GABA-A receptor antagonist picrotoxin and NMDA does not attenuate the effect of NMDA on electrically stimulated accumbal dopamine release. Therefore, the action of NMDA is unlikely to be mediated through GABA-A receptors. However, GABA-B receptors provide another mechanism through which the attenuation of dopamine release by NMDA may occur,^31−33^ particularly as there is evidence for GABA-B heteroreceptors on accumbal dopamine terminals.^34^ GABA-B agonists decrease accumbal dopamine release *in vivo*(35) and in rat^36^ and mouse^32^ brain slices *in vitro*. Therefore, GABA-B receptors do regulate accumbal dopamine release, but whether this mediates the effect of NMDA remains unclear. Moreover, the effect of pretreatment with the noncompetitive NMDA receptor antagonist, phencyclidine, on the GABA-B mediated modulation of stimulated dopamine release^36^ is consistent with a potential role of GABA-B mechanisms mediating the attenuating effect of NMDA on stimulated dopamine release, although this is not equivocal and warrants further investigation.

Currently, eight different mGluRs have been identified, which are subdivided into three groups, group I (mGluRs 1 and 5), group II (mGluRs 2 and 3), and group III (mGluRs 4, 6, 7, and 8), and have been shown to be present in NAc.^37^

Group I mGluRs are G_q_-protein couples and mainly located postsynaptically where they generally exert an excitatory action.^37,38^ Groups II and III, on the other hand, are G_i/o_-protein coupled and negatively regulate neurotransmitter release:^37,39,40^ while group III mGluRs are found pre- and postsynaptically and on glial cells, group II mGluRs are mainly located on presynaptic terminals,^37,39,40^ including on dopamine terminals where they negatively modulate dopamine release.^37^ Notably, presynaptic group II mGluRs are present both in the synapse, and outside the synapse, toward the axonal part, spatially removed from the transmitter release site^37,41^ where they respond to extra-synaptic “spillover” glutamate.^42^

Microdialysis studies *in vivo* indicated that activation of groups II and III but not group I mGluRs decreased dopamine release in NAc,^43,44^ although there was some indication that the effect of group II agonists was biphasic, initially reducing dopamine release before increasing it again.^43^ Moreover, schizophrenia-like behavioral changes induced by ketamine in animal models are somewhat reversed following the application of a group II agonist.^45^ Importantly, Yavas and Young^14^ showed that the broad spectrum mGluR antagonist, with little selectivity between groups I, II, and III, reduced the attenuation of electrically stimulated dopamine release by NMDA in NAc of rat brain slices *in vitro*, suggesting a key role of mGluRs in the attenuating action of NMDA. Therefore, there is strong evidence for mGluR modulating dopamine release in NAc, which could mediate the NMDA-induced effects, of which mGluR-II’s are most likely, given their negative modulatory nature, inhibiting dopamine release, and their extra-synaptic localization.

Therefore, cholinergic, GABA-ergic, and mGluR systems all exert inhibitory control over local dopamine release in NAc. The experiments used FSCV in rat brain slices *in vitro* to ascertain which, if any, of these mechanisms mediate attenuation of electrically stimulated dopamine release by NMDA.

## Results and Discussion

Electrical stimulation evoked a consistent release of dopamine from brain slices *in vitro*, which represented a released concentration of 0.204 ± 0.011 μM (*n* = 112 slices). There were no significant differences in baseline release between treatment groups. Similarly, there were no significant differences in any of the parameters measured between slices taken from male and female animals (see Supporting Information), so the data for both sexes were pooled.

In nondrug treated slices the stimulated release remained stable across the duration of the experiment comprising 14 or 18 stimulations, and in each experimental condition NMDA (30 μM) caused an attenuation of the stimulated dopamine release, to around 50% of baseline stimulation levels, consistent with previous data.^14^

### Experiment 1: Effect of Cholinergic Antagonists

The nicotinic receptor antagonist, dihydro-β-erythroidine (DHβE) (1 μM), applied alone caused a substantial increase in stimulated dopamine release, as has been reported previously.^14,46,47^ Although data from Yavas and Young^14^ suggested that DHβE may block the effects of NMDA, interpretation was impeded by the fact that DHβE itself increases the stimulated release. For this reason an extended protocol was used in the current experiments such that DHβE was applied alone for 12 min (4 stimulations), to establish a new baseline release in the presence of the antagonist, and then NMDA was added along with DHβE for a further 12 min (4 stimulations). DHβE alone caused a rise in stimulated dopamine release to a maximum of approximately 150% of baseline, consistent with previous findings.^14^

**Figure 1:**
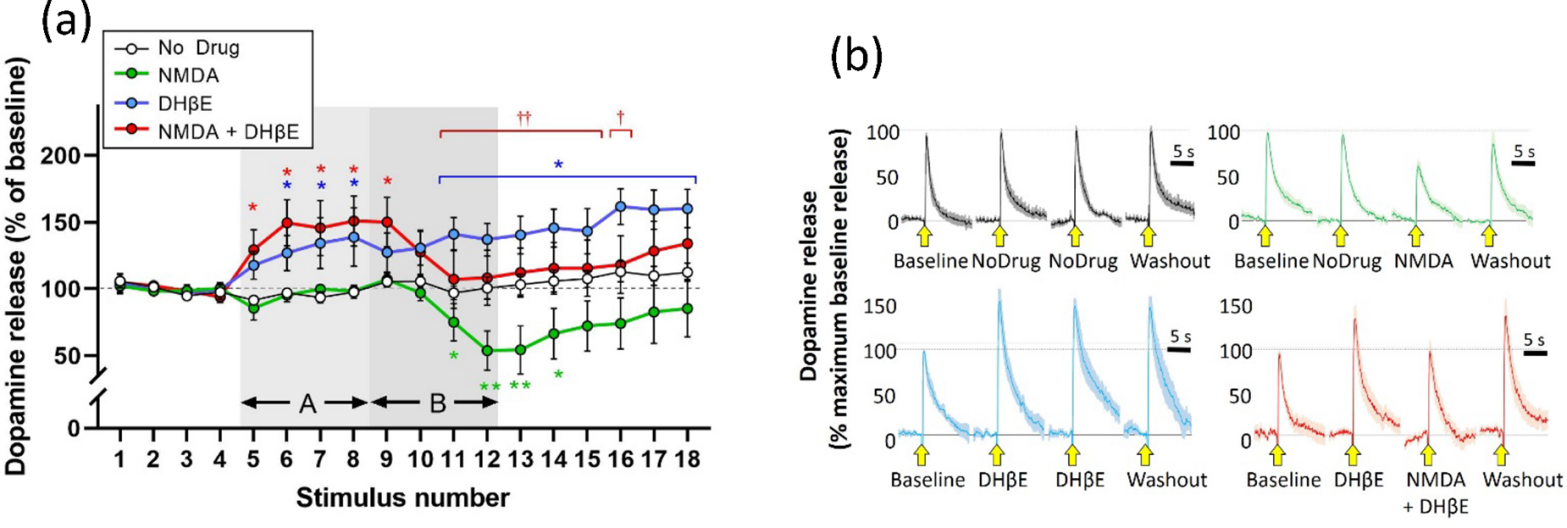
Effect of DHβE (1 μM) on attenuation of stimulated dopamine release caused by NMDA (30 μM). (a) Electrically stimulated dopamine release over repeated stimulations at 3 min intervals, presented as the mean ± SEM percentage of release during the baseline period (stimulations S1–S4). DHβE was applied in the superfusate for 12 min during stimulations S5–S12 (light gray panel, A), NMDA was applied for 12 min during stimulations S9–S14 (dark gray panel, B). *p < 0.05; **p < 0.01: significant difference from the no drug condition (post hoc Fisher’s LSD based on significant interaction in three-way ANOVA). ^†^*p* < 0.05; ^††^*p* < 0.01: significant difference from DHβE baseline immediately prior to NMDA + DHβE application (NMDA + DHβE condition (red line)); *n* = 8 per treatment condition. (b) Mean ± SEM responses during baseline (S4), drug A (S8), drug B (S12), and washout (S18) in the four treatment conditions. Stimulation application is indicated by the yellow arrow. Data are normalized to the maximum response during the baseline recording (S4): *n* = 8 per treatment condition.

When NMDA (30 μM) was added concomitantly with DHβE (1 μM), there was an attenuation of stimulated release down to near baseline levels, representing a reduction of approximately 50% of baseline (Figure 1). This reduction is comparable to the reduction seen when NMDA was given in the absence of DHβE (Figure 1).

Statistical analysis using a mixed-design three-way ANOVA showed main effects of stimulus (*F*(2.727, 76.36) = 3.580; *p* = 0.0208) and of DHβE (*F*(1, 28) = 6.931; *p* = 0.0136) but not of NMDA (*F*(1, 28) = 0.1849; *p* = 0.6705). The two-way interaction between stimulus and NMDA was significant (*F*(17, 476) = 2.873; *p* = 0.0001) as was the stimulus × DHβE (*F*(17, 476) = 2.386; *p* = 0.0015), but neither the remaining two-way interaction (NMDA × DHβE (*F*(1, 28) = 0.000 385 7; *p* = 0.9845)) or the three-way interaction (*F*(17, 476) = 0.4751; *p* = 0.9636) was significant.

Post hoc analysis (Fisher’s LSD) showed that during the application of DHβE alone (stimulations S5–S8) the stimulated release of dopamine was significantly augmented. In the DHβE alone condition, this augmentation was sustained through stimulations S9–S12, during which DHβE alone continued to be applied, and through stimulations S13– S18, during the washout period where the tissue was superfused with artificial cerebrospinal fluid (aCSF). NMDA alone applied during stimulations 9–12 caused a significant reduction in the stimulated release. In addition, when NMDA was applied alongside DHβE, during stimulations S9–S12, there was a significant reduction in stimulated release, resembling the reduction seen when NMDA was applied alone during this period, although starting from an elevated baseline (Figure 1).

The muscarinic AChR antagonist scopolamine (1 μM) alone had no effect of stimulated dopamine release: therefore, the standard 14 stimulus procedure was used rather than the extended 18 stimulus protocol. Scopolamine had no effect on the attenuation of stimulated dopamine caused by NMDA.

Statistical analysis using a mixed-design three-way ANOVA showed a main effect of NMDA (*F*(1, 28) = 6.713; *p* = 0.0150) but not of stimulus (*F*(2.831, 79.27) = 1.227; *p* = 0.3050) or of scopolamine (*F*(1, 28) = 0.2444; *p* = 0.6249). The stimulus × NMDA interaction was significant, but the other two two-way interactions (stimulus × scopolamine, *F*(13, 364) = 0.7865; *p* = 0.6747; NMDA × scopolamine, *F*(1, 28) = 0.0642; *p* = 0.8018) and the three-way interaction (*F*(13, 364) = 0.5006; *p* = 0.9239) were nonsignificant.

**Figure 2:**
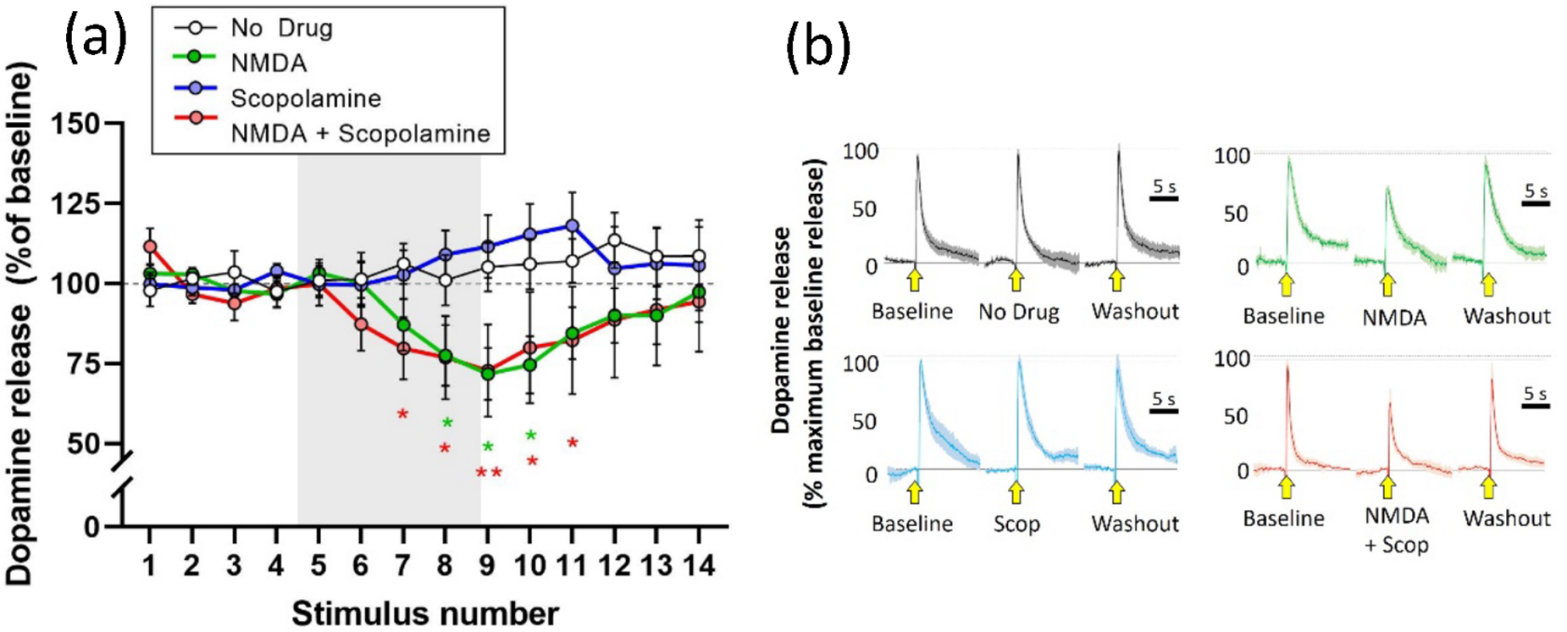
Effect of scopolamine (1 μM) on attenuation of stimulated dopamine release caused by NMDA (30 μM). (a) Electrically stimulated dopamine release over repeated stimulations at 3 min intervals, presented as the mean ± SEM percentage of release during the baseline period (stimulations S1–S4). Drugs were applied in the superfusate, either alone or in combination, for 12 min during stimulations S5–S12 (gray panel). *p < 0.05; **p < 0.01: significant difference from the no drug condition (post hoc Fisher’s LSD based on significant interaction in three-way ANOVA); *n* = 8 per treatment condition. (b) Mean ± SEM responses during baseline (S4), drug (S8), and washout (S14) in the four treatment conditions (Scop = scopolamine). Stimulation application is indicated by the yellow arrow. Data are normalized to the maximum response during the baseline recording (S4): *n* = 8 per treatment condition.

Post hoc analysis (Fisher’s LSD) confirmed the significant attenuation of stimulated dopamine release by NMDA. Scopolamine applied alone had no significant effect, nor did it have any significant effect on the attenuation of stimulated release caused by NMDA (Figure 2).

The summary data (Figure 6) emphasize the point that NMDA attenuated the stimulated dopamine release in the presence of either DHβE or scopolamine.

### Experiment 2: Effects of GABAergic Antagonists

Previous studies^14^ have shown that the GABA-A antagonist picrotoxin failed to block the attenuation of stimulated release caused by NMDA.

We extended this by testing whether blockade of GABA-B receptors affected the NMDA-induced attenuation. As with DHβE, the GABA-B antagonist CGP 54626 (1 μM) caused a marked increase in stimulated dopamine release when applied alone, consistent with previous reports,^36^ and therefore required the extended (18 stimulus) protocol to establish a new baseline release in the presence of the antagonist, before applying NMDA. In these conditions, NMDA continued to reduce stimulated dopamine release, even in the presence of the GABA-B antagonist.

Statistical analysis using a mixed-design three-way ANOVA showed main effects of stimulus, (*F*(2.307, 73.83) = 6.347; *p* = 0.0018), NMDA (*F*(1, 32) = 8.674; *p* = 0.0060), and CGP 54626 (*F*(1, 32) = 16.02; *p* = 0.0003). All two-way interactions and the three-way interaction were also significant (stimulus × NMDA, *F*(17, 544) = 9.064; *p* < 0.0001; stimulus × CGP 54626 (*F*(17, 544) = 7.404; *p* < 0.0001; (NMDA × CGP 54626 (*F*(1, 32) = 6.188; *p* = 0.0183); three-way interaction (*F*(17, 544) = 3.718; *p* < 0.0001)).

**Figure 3:**
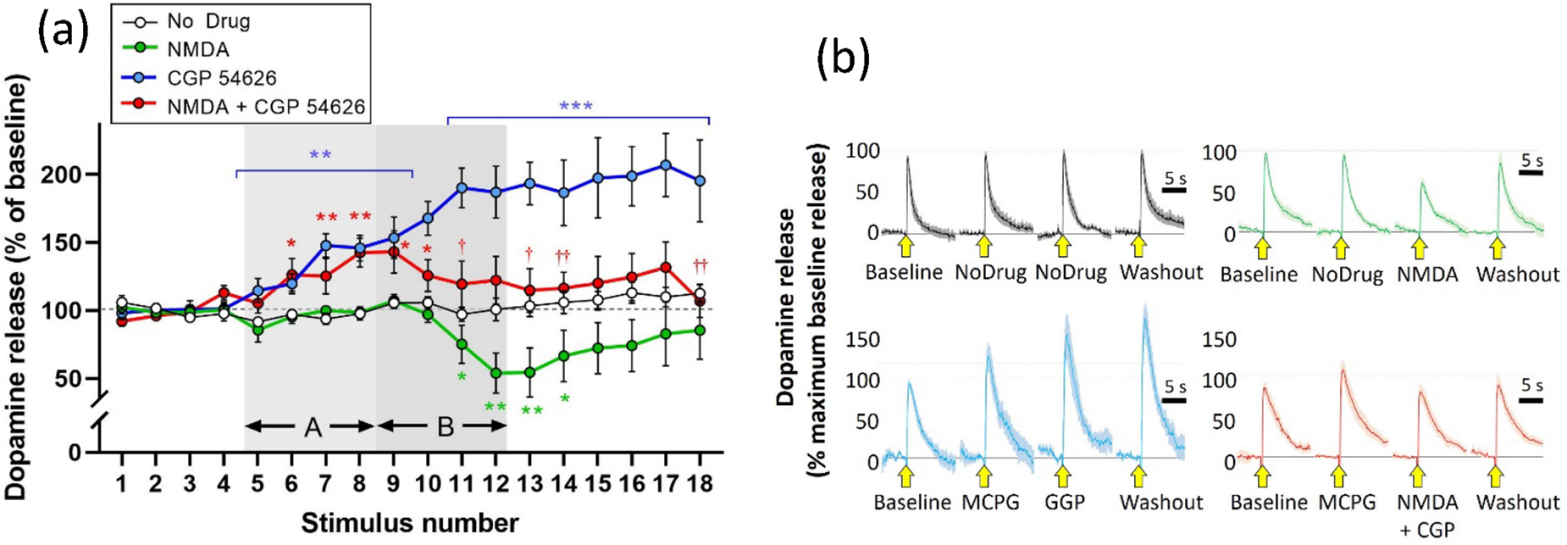
Effect of CGP 54626 (1 μM) on attenuation of stimulated dopamine release caused by NMDA (30 μM). (a) Electrically stimulated dopamine release over repeated stimulations at 3 min intervals, presented as mean ± SEM percentage of release during the baseline period (stimulations S1–S4). CGP 54626 was applied in the superfusate for 12 min during stimulations S5–S12 (light gray panel, A), and NMDA was applied for 12 min during stimulations S9–S14 (dark gray panel, B). *p < 0.05; **p < 0.01: significant difference from the no drug condition (post hoc Fisher’s LSD based on significant interaction in three-way ANOVA). ^†^*p* < 0.05; ^††^*p* < 0.01: significant difference from CGP 54626 baseline immediately prior to NMDA + CGP 54626 application (NMDA + CGP 54626 condition (red line)); *n* = 8 per treatment condition. (b) Mean ± SEM responses during baseline (S4), drug A (S8), drug B (S12), and washout (S18) in the four treatment conditions (CGP = CGP 54626). Stimulation application is indicated by the yellow arrow. Data are normalized to the maximum response during the baseline recording (S4): *n* = 8 per treatment condition.

Post hoc analysis (Fisher’s LSD) showed a significant increase in stimulated dopamine release when CGP 54626 was applied alone during stimulations S5–S8, although the onset was delayed and only reached statistical significance from stimulus S6. Where CGP 54626 application continued alone over the next four stimulations (S9–12) the stimulated response continued to rise. As previously, NMDA applied during stimulations S9–S12 caused a significant attenuation on stimulated dopamine release, although the effect was delayed by around 6 min, only showing up in stimulations S11–S14. When NMDA was applied in the presence of CGP 54626, the attenuation remained intact, albeit that it started from a raised baseline and showed a significant difference from CGP 54626 alone (Figure 3). As before, the summary data (Figure 6) clearly indicate that CGP 54626 does not affect the attenuation of stimulated dopamine release caused by NMDA.

### Experiment 3: Effect of mGluR Antagonists

The broad spectrum (groups I, II, and III) mGluR antagonist α-methyl-4-carboxyphenylglycine (MCPG: 100 μM) had no effect of stimulated dopamine release when applied alone but completely abolished the attenuation of stimulated release caused by NMDA (30 μM).

**Figure 4:**
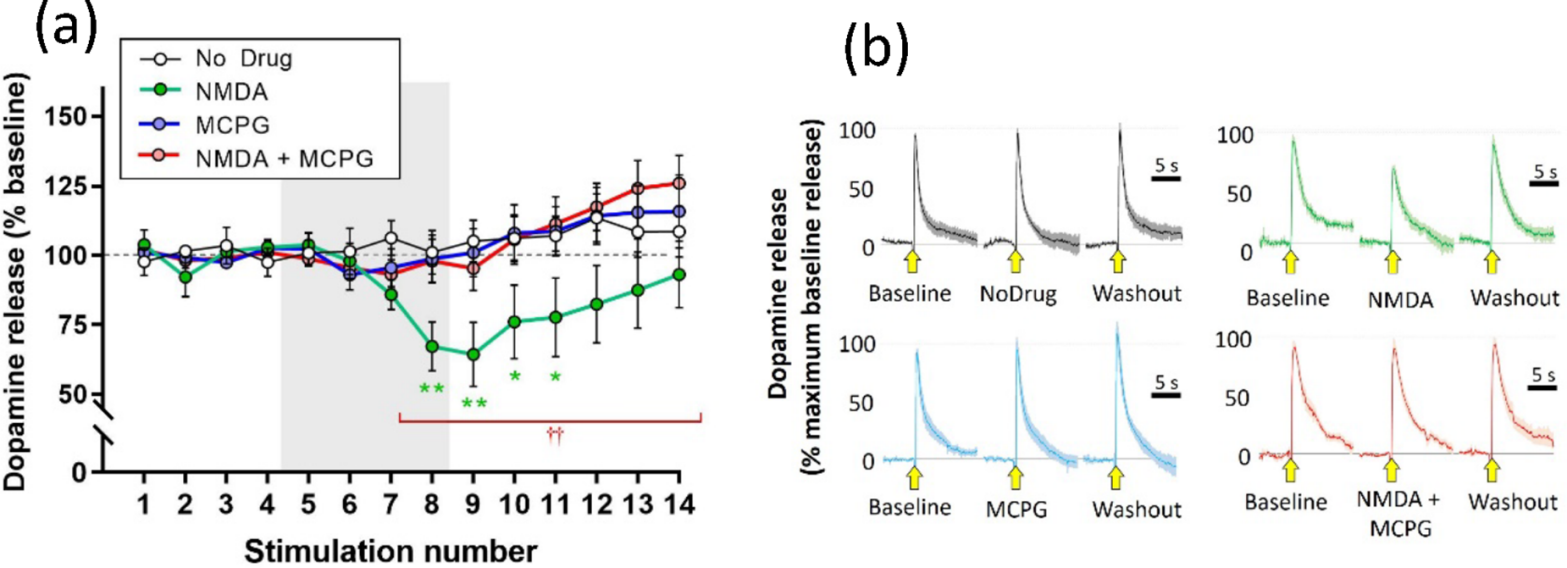
Effect of MCPG (100 μM) on attenuation of stimulated dopamine release caused by NMDA (30 μM). (a) Electrically stimulated dopamine release over repeated stimulations at 3 min intervals, presented as mean ± SEM percentage of release during the baseline period (stimulations S1–S4). Drugs were applied in the superfusate, either alone or in combination, for 12 min during stimulations S5–S12 (gray panel). *p < 0.05; **p < 0.01: significant difference from the no drug condition: ^††^*p* < 0.01: significant difference between NMDA application in the absence or presence of MCPG (post hoc Fisher’s LSD based on significant interaction in three-way ANOVA): *n* = 8 per treatment condition. (b) Mean ± SEM responses during baseline (S4), drug (S8), and washout (S14) in the four treatment conditions. Stimulation application is indicated by the yellow arrow. Data are normalized to the maximum response during the baseline recording (S4): *n* = 8 per treatment condition.

Statistical analysis using a mixed-design three-way ANOVA showed a main effect of stimulus (*F*(2.268, 63.51) = 3.935; *p* = 0.0202) but not of NMDA (*F*(1, 28) = 3.169; *p* = 0.0859) or MCPG (*F*(1, 28) = 0.6277; *p* = 0.4349). The two-way interaction between stimulus and NMDA (*F*(13, 364) = 2.374; *p* = 0.0046) and the three-way interaction (*F*(13, 364) = 2.585; *p* = 0.0019) were significant, but the remaining two-way interactions were nonsignificant (stimulus × MCPG *F*(13, 364) = 1.264; *p* = 0.2323: NMDA × MCPG, *F*(1, 28) = 3.713; *p* = 0.0642). Post hoc analysis (Fisher’s LSD) confirmed the significant attenuation of stimulated dopamine release by NMDA during stimulations S8–S11. MCPG applied alone had no significant effect but completely reversed the attenuation of stimulated release caused by NMDA (Figure 4)

Subsequent experiments used the selective group II antagonist, LY 341495 (1 μM). This also had no effect on stimulated dopamine when applied alone but abolished the attenuation of stimulated release caused by NMDA (30 μM).

Statistical analysis using a mixed-design three-way ANOVA showed a main effect of stimulus (*F*(2.386, 66.80) = 3.048; *p* = 0.0454) but not of NMDA (*F*(1, 28) = 3.363; *p* = 0.0773) or LY 341495 (*F*(1, 28) = 1.219; *p* = 0.2789). The two-way interaction between stimulus and NMDA (*F*(13, 364) = 2.414; *p* = 0.0039) and the three-way interaction (*F*(13, 364) = 2.392; *p* = 0.0043) were significant, but the remaining two-way interactions were nonsignificant (stimulus × LY 341495, *F*(13, 364) = 1.573; *p* = 0.0906: NMDA × LY 341495, *F*(1, 28) = 3.139; *p* = 0.0873).

**Figure 5:**
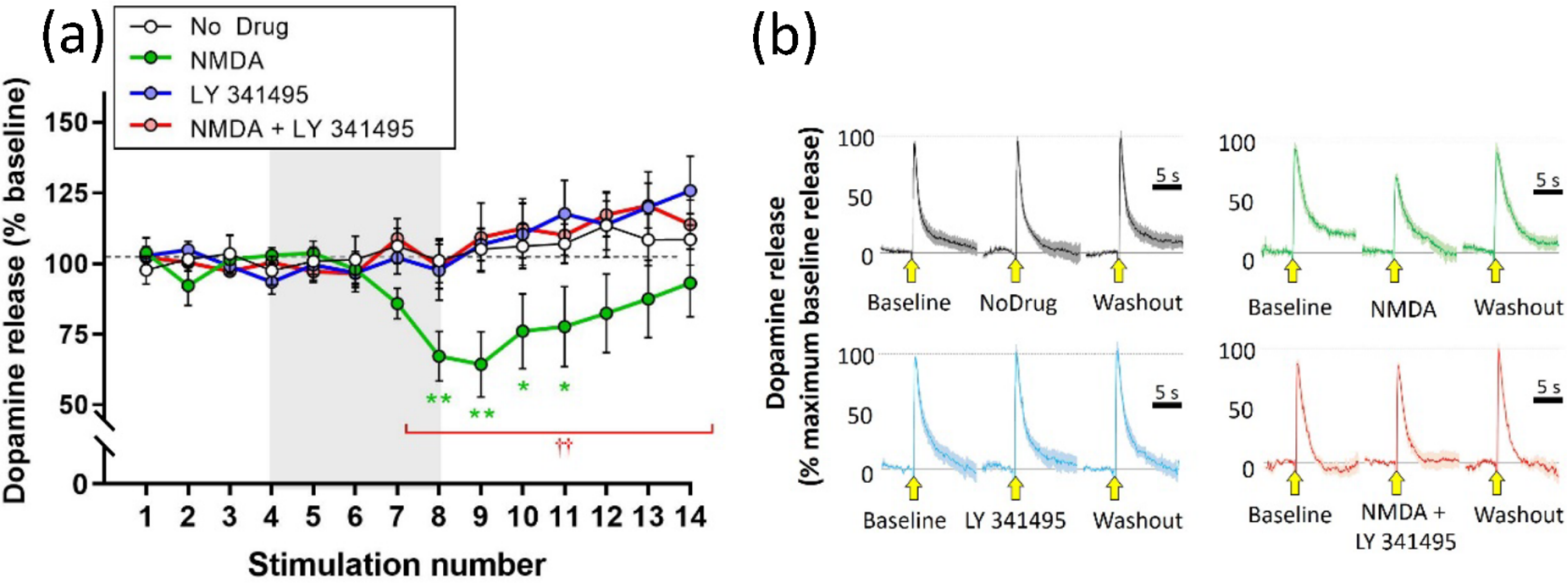
Effect of LY 341495 (1 μM) on attenuation of stimulated dopamine release caused by NMDA (30 μM). (a) Electrically stimulated dopamine release over repeated stimulations at 3 min intervals, presented as mean ± SEM percentage of release during the baseline period (stimulations S1–S4). Drugs were applied in the superfusate, either alone or in combination, for 12 min during stimulations S5–S12 (gray panel). *p < 0.05; **p < 0.01: significant difference from the no drug condition; significant difference between NMDA application in the absence or presence of LY 341495 (post hoc Fisher’s LSD based on significant interaction in three-way ANOVA): *n* = 8 per treatment condition. (b) Mean ± SEM responses during baseline (S4), drug (S8), and washout (S14) in the four treatment conditions. Stimulation application is indicated by the yellow arrow. Data are normalized to the maximum response during the baseline recording (S4): *n* = 8 per treatment condition.

Post hoc analysis (Fisher’s LSD) showed that NMDA significantly attenuated the stimulated dopamine release during stimulations S8–S11. LY 341495 alone had no effect on stimulated dopamine but completely abolished the effect of NMDA (Figure 5)

**Figure 6:**
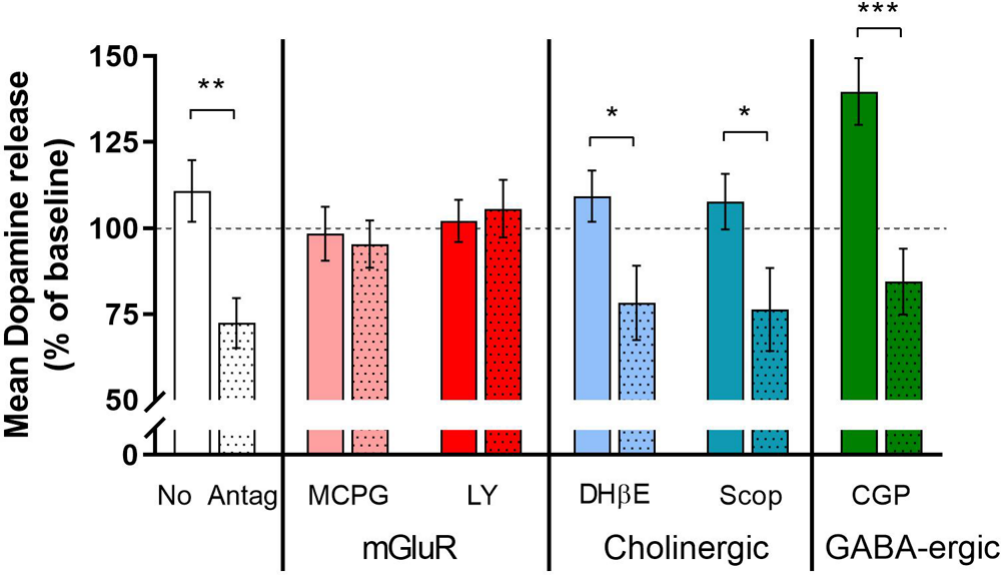
Summary of the effects of antagonists on the attenuation of electrically stimulated dopamine caused by NMDA: the effects of the mGluR antagonists, MCPG (100 μM) and LY 341495 (LY, 1 μM) (red bars); the cholinergic antagonists, DHβE (1 μM) and scopolamine (Scop, 1 μM) (blue bars); and the GABA-B antagonist, CGP 54626 (CGP, 1 μM) (green bars) alone (open bars) and of NMDA (30 μM) plus the respective antagonist (shaded bars). Noncolored bars depict the attenuation caused by NMDA with no antagonist present. All data are the percentage of the mean stimulated release in the three stimulations before the application of the respective drugs. *p < 0.05; **p < 0.01: significant difference between antagonist alone and agonist + antagonist (*t* test).

The summary data (Figure 6) emphasize the point that NMDA did not attenuate the stimulated dopamine release in the presence of either MCPG or LY 341495.

In summary, the results showed that repeated electrical stimulation at 3 min intervals evoked a reliable release of dopamine, which remained stable over the duration of the experiment, comprising either 14 stimulations (39 min) or 18 stimulations (51 min). NMDA (30 μM) caused a reliable attenuation of stimulated dopamine release to around 50% of baseline stimulation, consistent with previous studies.^14^ Neither the nicotinic AChR antagonist DHβE (1 μM) nor the muscarinic AChR antagonist scopolamine (1 μM), at concentrations previously shown to be effective at blocking cholinergic actions, affected the NMDA-evoked attenuation. Similarly, the GABA-B receptor antagonist CGP 54626 (1 μM), at a concentration known to block the GABA-B function,^36^ did not reverse the NMDA-evoked attenuation. However, the broad-spectrum (group I/II/III) mGluR antagonist MCPG (100 μM) and the specific group II mGluR antagonist LY 341495 (1 μM) completely reversed the effect of NMDA.

It is known that dopamine release from mesolimbic neurones is under modulatory control at the level of the terminals in NAc from many neurotransmitters, including glutamate, GABA, and acetylcholine. By making our recordings in brain slices, we can be confident that we are measuring local, intra-accumbens modulatory control, as long-loop network connections are absent in slices. We previously showed that NMDA (30 μM) caused an attenuation of electrically stimulated dopamine release in NAc slices,^14^ which is thought to be mediated through an inhibitory intermediary rather than by a direct action on the dopamine terminals.

Cholinergic interneurons have been shown to exert strong modulatory control over dopamine release from mesolimbic terminals in NAc, through both nicotinic and muscarinic receptors,^19,30,46−48^ which are widely expressed in mesolimbic dopamine neurones in NAc.^30,49^ DHβE, when given alone, evoked an augmentation of the stimulated release of dopamine, similar to that seen previously,^14,50^ consistent with cholinergic inhibition of dopamine release through local nicotinic receptor mediated mechanisms. However, DHβE did not reduce the attenuation of stimulated dopamine release caused by NMDA: in slices superfused with DHβE, NMDA still caused an attenuation of the stimulated release of similar magnitude to that seen in the absence of DHβE, albeit from an elevated baseline (Figures 1 and 6).

Similarly, scopolamine had no effect on the attenuation of stimulated release caused by NMDA. Since previous studies have shown that the doses of these drugs used here were sufficient to block nicotinic and muscarinic receptors, respectively (ref (14) and unpublished data), we can be confident that the lack of effect of either antagonist on the attenuation of stimulated dopamine release caused by NMDA does indeed reflect that the NMDA effect is not mediated through cholinergic mechanisms.

GABA is also known to exert inhibitory control over mesolimbic dopamine release at the terminals in NAc.^36^ Both GABA-A and GABA-B receptors are located on dopamine terminals in NAc,^51^ where they exert a modulatory influence over mesolimbic dopamine release,^51,52^ so it is plausible that NMDA modulation of dopamine release may be mediated through such GABA mechanisms. Previous studies have shown that the GABA-A receptor antagonist picrotoxin does not diminish the attenuation of stimulated dopamine release caused by NMDA;^14^ therefore the current experiments focused on GABA-B receptors. As with DHβE and consistent with previous experiments,^36^ the GABA-B receptor antagonist CGP 54626 caused an increase in stimulated dopamine release when applied alone. We therefore used the extended protocol, where a new baseline was established in the presence of the antagonist before application of the NMDA, facilitating dissociation of the effects of the two drugs. The NMDA attenuation of stimulated dopamine release remained intact in the presence of CGP 54626. As with the cholinergic drugs, the dose of CGP 54626 used has previously been shown to reverse the effects GABA-B agonists,^36^ so we can be confident that blockade occurred here and that the effect of NMDA is not mediated through a GABAergic intermediary.

Accumbal dopamine release is also modulated by mGluR at the level of the terminals (e.g., ref (43)), and both direct actions on the dopamine terminals and indirect actions via intermediary neurones are plausible, since mGluRs have been visualized on both presynaptic and postsynaptic elements (see Introduction). Initial experiments using a broad spectrum mGluR antagonist, MCPG, which is equipotent at group I and group II mGluRs and also has some effect on group III, showed that it completely blocked the attenuation of dopamine release caused by NMDA, confirming previous findings.^14^

Group II mGluRs have a negative modulatory effect of transmitter release^39,40^ and, as such, seemed a likely candidate to mediate the mGluR action. Moreover, studies using the selective group II antagonist LY 341495 showed that blocking group II mGluR also abolished the attenuation of stimulated dopamine release caused by NMDA. Evidence suggests that group II mGluRs are located on dopamine terminals but are outside the synapse, toward the axonal part of the terminal, and are activated by extra-synaptic, “spillover” glutamate.^38,41^ Moreover, NMDA receptors are also found extra-synaptically on glutamate terminals: these receptors are generally slower than their intrasynaptic counterparts but with higher affinity.^53,54^ They regulate neuronal membrane potential, increasing excitability and further enhancing glutamate release from the terminal.^55,56^ The increased glutamate release, in turn, leads to more spillover which activates receptors over a wider volume, including the group II mGluRs on surrounding dopamine terminals, leading to inhibition of dopamine release. This provides a plausible mechanism through which NMDA can attenuate electrically stimulated dopamine release through group II mGluR-controlled signaling. It is pertinent to note that, in particular, high frequency stimulation increases the spillover of glutamate:^56^ this is consistent with the findings from the current study, which employed high frequency (60 Hz) stimulation, and also with preliminary data (Supporting Information) which showed no effect of NMDA on electrically stimulated dopamine release at low frequency stimulation.

The glutamate theory of schizophrenia posits a core deficit is in NMDA receptor function,^10−13^ which leads to downstream effects on transmitter systems, including glutamate/dopamine dysregulation.^5^ Moreover, mGluR involvement in schizophrenia has been posited^57,58^ possibly through disrupted interactions between NMDA and mGluR.^59^ More specifically, group II mGluR agonists reverse changes in behaviors resembling schizophrenia in animal models of schizophrenia,^59−61^ leading to these drugs being considered as potential novel antipsychotic drugs.^62,63^ The results shown here provide a plausible mechanism through which NMDA/dopamine dysregulation in schizophrenia may involve group II mGluR mechanisms, giving further impetus for studying this class of drugs as potential therapeutic agents.^62,63^

In conclusion, these experiments aimed to elucidate the mechanism that mediates the attenuation of electrically stimulated dopamine release caused by NMDA in NAc. Three possible candidates for this mechanism were investigated: cholinergic, GABA-ergic, and mGluR. The findings showed that neither cholinergic nor GABA-ergic antagonists have any effect on the change caused by NMDA but that mGluR antagonists, particularly group II, completely abolish the NMDA-evoked effect. This indicates that the attenuation of electrically stimulated dopamine release caused by NMDA in NAc is mediated through group II mGluR, but not cholinergic or GABAergic mechanisms, probably through a direct inhibitory effect of these receptors located extra-synaptically on dopamine terminals.

## Methods

### Animals

Male and female rats (Charles River, U.K.; 100–150 g) were housed in independently ventilated, double-deck Plexiglas cages (46W cm × 40D cm × 40H cm; Techniplast, U.K.) in groups of four to six animals at the University of Leicester Preclinical Research Facility. Animals were maintained under standard laboratory conditions as temperature (21 ± 2 °C), humidity (55% ± 10%), and lighting (12 h light/dark cycle, lights on at 07:00) were held constant. Animals could access food (LabDiet 5LF5, IPS Ltd., U.K.) and water *ad libitum*. This project received ethical approval from the University of Leicester Ethical Committee (AWERB/2019/69).

### Procedure

The procedure was similar to that previously described.^14,23,36^ Animals underwent cervical dislocation, and the brain was removed and placed into ice-cold aCSF, comprising (mM), NaCI (126.0), KCI (2.0), KH_2_PO_4_ (1.4), MgSO_4_ (2.0), NaHCO_3_ (26.0), CaCI_2_ (2.4), glucose (10.0). Coronal slices (400 μm) containing NAc were cut using a vibrating microtome (752 M Vibroslice, Campden Instruments, U.K.). Each brain provided three to four slices containing NAc: these were cut along the midline to provide two single hemisphere slices from each full slice. With one hemislice used for each experimental condition, it allowed six to eight experimental conditions to be tested from each brain. Slices were then incubated in oxygenated (95% O_2_/5% CO_2_) aCSF at a temperature of 21 ± 2 °C for 60 min to recover from the trauma of slicing.

For recording, slices were transferred to the recording chamber and superfused continuously with oxygenated aCSF (31 ± 2 °C; 2.0 mL/min delivered via a Gilson Minipuls 3 peristaltic pump). Slices equilibrated for 30 min before a concentric bipolar tungsten stimulating electrode (CBARC75, FHC Inc., Bowdoin, USA) and a carbon fiber recording electrode, custom built in the lab as described by Clark et al.,^64^ were placed into the NAc, with the recording electrode approximately 0.5 mm away from the stimulating electrode.

A triangular waveform (−0.4 to +1.3 to −0.4 V; 400 V/s relative to Ag/AgCl reference electrode) was applied at a frequency of 10 Hz using Demon voltammetry software,^65^ connected to a Chem-Clamp potentiostat with a 5 MΩ headstage (Dagan Corporation, USA). The current generated was recorded, and dopamine oxidation was measured on the forward scan at approximately +0.6 V, in the background subtracted signal (Demon Voltammetry software^65^).

Slices were stimulated (10 × 1 ms pulses; 800 μA; 60 Hz delivered via a constant current stimulus isolator: Iso-Flex; AMP Instruments) at 3 min intervals, during a 15 s recording period, with the stimulus onset 5 s after the start of the recording. For experiments 1b and 3, 14 stimulus trains were administered at 3 min intervals with the complete session lasting 39 min. In the no drug (control) condition, tissue was superfused with aCSF throughout. For drug conditions, slices were perfused with aCSF for the first 9 min (four stimulations; S1–S4) and then drug was applied in the superfusate for 12 min (stimulations S5–S8). The drug was then removed, and slices superfused with aCSF again for the remaining 18 min of the experiment (stimulations S9–S14)

For experiments 1a and 2, where the antagonist alone had an effect opposite to the NMDA effect, an extended 18-stimulation protocol was utilized. Here, 18 stimulation trains were applied to the tissue at 3 min intervals in a session lasting for a total of 51 min. As before, in the no drug (control) condition, aCSF was perfused throughout the session for 51 min. In the NMDA alone condition, aCSF was perfused for 24 min, from stimulations S1–S8, before NMDA (30 μM) was applied in the superfusate for 12 min (stimulations S9–S12), after which the superfusate was returned to aCSF for 18 min (stimulations S13–S18). Where antagonists were used, these were applied in the superfusate for 24 min, after the initial 9 min baseline recording period (stimulations S5–S12), before returning to aCSF for 18 min (stimulations S13–S18). In experiments testing the effect of the antagonist on NMDA following 12 min of application of the antagonist alone (stimulations S5–S8), NMDA was applied concomitantly with the antagonist for 12 min (stimulations S9–S12), before the final 18 min with aCSF alone (stimulations S13–S18).

#### Experiment 1: Effect of Cholinergic Antagonists

(a) Nicotinic Antagonist, DHβE. After 9 min baseline superfusion with aCSF, the DHβE and NMDA + DHβE treatment groups received DHβE (1 μM) alone for 12 min (stimulations S5–S8), while the no drug and NMDA groups continued to receive aCSF. During the next 12 min (stimulations S9–S12) either DHβE alone (DHβE group), NMDA (30 μM) alone (NMDA group), or a combination of the two (NMDA + DHβE group) was applied in the superfusate. Finally, all groups were returned to aCSF superfusion for the remaining 18 min of recording (stimulations S13–S18). The no drug condition received aCSF throughout the full 51 min of recording.

(b) Muscarinic Antagonist, Scopolamine. After 9 min baseline superfusion with aCSF, either NMDA (30 μM), scopolamine (1 μM), or the combination of NMDA (30 μM) and scopolamine (1 μM) was applied in the superfusate for 12 min (stimulations S5–S8), before returning to aCSF superfusion for 18 min.

#### Experiment 2: Effect of GABAergic Antagonists

GABA-B Antagonist, CGP 54626. After 9 min baseline superfusion with aCSF, the CGP 54626 and NMDA + CGP 54626 treatment groups received CGP 54626 (1 μM) alone for 12 min (stimulations S5–S8), while the no drug and NMDA groups continued to receive aCSF. During the next 12 min (stimulations S9–S12) either CGP 54626 alone (CGP 54626 group), NMDA (30 μM) alone (NMDA group), or a combination of the two (NMDA + CGP 54626 group) was applied in the superfusate. Finally all groups were returned to aCSF superfusion for the remaining 18 min of recording (stimulations S13–S18). The no drug condition received aCSF throughout the full 51 min of recording.

#### Experiment 3: Effect of mGluR Antagonists

(a) Group I, II and III mGluR antagonist, MCPG. After 9 min baseline superfusion with aCSF, either NMDA (30 μM), MCPG (100 μM), or the combination of both was applied in the superfusate for 12 min (stimulations S5–S8), before returning to aCSF superfusion for 18 min.

(b) Group II mGluR-II Antagonist, LY 341495. After 9 min baseline superfusion with aCSF, either NMDA (30 μM), LY 341495 (1 μM), or the combination of both was applied in the superfusate for 12 min (stimulations S5–S8), before returning to aCSF superfusion for 18 min.

### Data Analysis

Demon Voltammetry and Analysis software^65^ recorded the Faradaic current following background subtraction of the signal. Dopamine (1 μM) calibration performed each day before experiments allowed the calculation of peak dopamine release concentration following each stimulation.

For all experiments, the first four stimulations (S1–S4) were used to calculate the mean baseline of electrically stimulated dopamine release. Then dopamine release recorded at all 14 (18 in the extended protocol) stimulations was calculated as the percentage of this mean baseline. Time course data from replications are shown as mean ± SEM percentages of this baseline stimulated dopamine release.

Summary data were also calculated for each portion of the recording by calculating the mean release during NMDA application as a percentage of the mean baseline in the three stimulations immediately before NMDA application (S2–S4 or S6–S8 in the extended protocol). Due to the delay in onset of the NMDA response, the first two stimulations after application of NMDA were disregarded, and the mean of the next three stimulations (S7–S9 or S11–S13 in the extended protocol) was designated as the NMDA response.

Statistical analysis was carried out using GraphPad Prism v9.0.0. Alpha level for analysis was 0.05. Time course and summary data were subjected to mixed-design three-way ANOVA (stimulus × NMDA × antagonist), where stimulus was a repeated measure, and NMDA and antagonist were between subject measures. Significant interactions were further investigated using Fisher’s LSD to ascertain differences between the experimental conditions during each stimulation (planned comparisons). Violations in sphericity were compensated using Greenhouse–Geisser correction.

For depiction of the profile of stimulated release, the raw (current vs time) data for stimulations S4 (baseline), S8 (drug), and S14 (washout) (or S4 (baseline), S8 (drug A), and S12 (drug B) and S18 (washout) for experiments using the extended protocol) were normalized to the maximum stimulation amplitude achieved in the baseline (S4) period. Pooled data (mean ± SEM) were then plotted for each condition.

### Chemicals and Drugs

MCPG, LY 341496 and CGP 45626 were supplied by Tocris (Bio-Techne, U.K.): all other drugs and chemicals for aCSF were supplied by Sigma-Aldrich (Poole, U.K.). All drugs were made up as 10 mM stock solutions in water except for CGP 45626, which was made up in DMSO. Aliquots were frozen (−20 °C) until use. On the day of experiments, a drug aliquot was thawed and diluted in aCSF to the appropriate working concentration. Drug concentrations were derived from previous work in our lab (refs (14), (23), and (36) and unpublished data].

## Supporting Information Available

Table S1 of data demonstrating that electrically stimulated dopamine release did not differ between sexes; Figure S1 of preliminary data demonstrating that NMDA does not attenuate dopamine release stimulated by low-frequency electrical stimulation (PDF)

## Abbreviations

aCSF: artificial cerebrospinal fluid
AChR: acetylcholine receptor
DhβE: dihydro-β-erythroidine
FSCV: fast-scan cyclic voltammetry
GABA: γ-aminobutyric acid
MCPG: α-methyl-4-carboxyphenylglycine
mGluR: metabotropic glutamate receptor
NAc: nucleus accumbens
NMDA: *N*-methyl-d-aspartate
PCP: phencyclidine
