# Supplementary material for: Attenuation of Stimulated Accumbal Dopamine Release by NMDA Is Mediated through Metabotropic Glutamate Receptors
Source: ACS Chem Neurosci. 2023 Apr 6;14(8):1449–58. doi: 10.1021/acschemneuro.2c00777 (PMC10119936; doi:10.1021/acschemneuro.2c00777)
Supplement: Supplementary file 1 — cn2c00777_si_001.pdf [file cn2c00777_si_001.pdf]

Attenuation of stimulated accumbal dopamine release by NMDA is mediated through metabotropic glutamate receptors

Felicity S.E. Spencer, Maria Glodkowska, Anna I Sebold, Ersin Yavas, Andrew M.J. Young

Attenuation of stimulated accumbal dopamine release by NMDA is mediated through metabotropic glutamate receptors

Felicity S.E. Spencer[1], Maria Glodkowska, Anna I Sebold, Ersin Yavas[2], Andrew M.J. Young*

School of Psychology and Vision Sciences, University of Leicester, Lancaster Road, Leicester, LE1 9HN, UK

Author Information.

[1]Felicity Spencer : School of Sport, Exercise and Rehabilitation Sciences, University of Birmingham, Edgbaston, Birmingham, B15 2TT

Maria Glodkowska : School of Psychology and Vision Sciences, University of Leicester, Lancaster Road, Leicester, LE1 9HN:

Anna Sebold : School of Psychology and Vision Sciences, University of Leicester, Lancaster Road, Leicester, LE1 9HN:

[2]Ersin Yavas : Department of Psychology, Bartın University, Bartın 74100, Turkey

Andrew Young : School of Psychology and Vision Sciences, University of Leicester, Lancaster Road, Leicester, LE1 9HN

Corresponding Author

Andrew Young : School of Psychology and Vision Sciences, University of Leicester, Lancaster Road, Leicester, LE1 9HN, U.K.

Telephone : (+44) 1162297111

e-mail : amjy1@le.ac.uk

Supplementary information

(1) Electrically-stimulated dopamine release does not differ across sexes

Method: Both male (58 slices) and female (54 slices) rats were used throughout this study (total 112), with n = 3 to 5 of each sex in each experimental condition.

Results: Electrical stimulation evoked a consistent rise in dopamine release measured during the baseline recording period, comprising stimulations S1 to S4, (0.204 ± 0.011 µM; n = 112 slices). Moreover, there was no significant difference between this baseline electrically-stimulated release, between males (0.205 ± 0.014 µM; n = 58 slices) and females (0.204 ± 0.164 µM; n = 54 slices: Table 1)

	Male	Female	**All animals**
All treatment groups	0.205 ± 0.014 (58)	0.204 ± 0.164 (54)	**0.204 ± 0.011 (112)**

Attenuation of stimulated accumbal dopamine release by NMDA is mediated through metabotropic glutamate receptors

Felicity S.E. Spencer, Maria Glodkowska, Anna I Sebold, Ersin Yavas, Andrew M.J. Young

No Drug (18 stimulations)	0.166 ± 0.020 (4)	0.207 ± 0.069 (4)	**0.176 ± 0.023 (8)**
NMDA (18 stimulations)	0.193 ± 0.029 (3)	0.189 ± 0.038 (5)	**0.190 ± 0.023 (8)**
CGP (18 stimulations)	0.179 ± 0.024 (4)	0.178 ± 0.037 (4)	**0.179 ± 0.021 (8)**
NMDA + CGP (18 stimulations)	0.234 ± 0.119 (4)	0.209 ± 0.078 (4)	**0.221 ± 0.066 (8)**
DHβE (18 stimulations)	0.216 ± 0.057 (4)	0.210 ± 0.013 (4)	**0.213 ± 0.027 (8)**
NMDA + DHβE (18 stimulations)	0.228 ± 0.070 (5)	0.175 ± 0.039 (3)	**0.208 ± 0.042 (8)**
No Drug (14 stimulations)	0.182 ± 0.027 (4)	0.172 ± 0.036 (4)	**0.177 ± 0.021 (8)**
NMDA (14 stimulations)	0.208 ± 0.035 (4)	0.212 ± 0.070 (4)	**0.210 ± 0.036 (8)**
Scopolamine (14 stimulations)	0.209 ± 0.046 (3)	0.228 ± 0.056 (5)	**0.221 ± 0.035 (8)**
NMDA + Scopolamine (14 stimulations)	0.180 ± 0.056 (4)	0.197 ± 0.032 (4)	**0.189 ± 0.030 (8)**
MCPG (14 stimulations)	0.206 ± 0.035 (4)	0.258 ± 0.062 (4)	**0.232 ± 0.038 (8)**
NMDA + MCPG (14 stimulations)	0.247 ± 0.099 (4)	0.262 ± 0.138 (4)	**0.255 ± 0.079 (8)**
LY 341495 (14 stimulations)	0.162 ± 0.025 (4)	0.188 ± 0.018 (4)	**0.175 ± 0.015 (8)**
NMDA + LY 341495 (14 stimulations)	0.227 ± 0.065 (4)	0.205 ± 0.123 (4)	**0.214 ± 0.067 (8)**

Table S1: Summary of electrically stimulated release of dopamine (μM) during the baseline recording period (stimulations S1 to S4). *Data are mean ± SEM release concentrations for males, females and both sexes combined, across all the experimental treatment groups, with number of slices per group shown in parentheses. No significant differences were seen between males and females in any experimental condition, nor between experimental* conditions.

(2) Effect of NMDA on electrically-stimulated dopamine release, using low frequency stimulation

Method: The procedure for slice preparation and electrode placement was as described for the main experiments. Briefly, coronal slices (400 μm) containing nucleus accumbens (NAc) were cut, and divided along the midline two provide two hemi-slices from each full slice, which were then allowed to equilibrate at room temperature for at least 30 min. A single hemi-slice was placed in the tissue chamber and superfused with artificial cerebrospinal fluid (aCSF; 33 ± 2 ˚C; 2 ml/min) for a further 30 min before recording started. A concentric bipolar stimulating electrode and a carbon fibre recording electrode were lowered into NAc, to lie just below the surface of the tissue and approximately 0.5 mm apart. Electrical stimulation was applied at low frequency (5 pulses, 10 Hz, 800 μA) at 3 min intervals, and the dopamine concentration was measured as the oxidation current at approximately 600 mV in the background subtracted current.

Results: Low frequency stimulation caused a reliable release of dopamine, but smaller amplitude than at high frequency (0.047 ± 0.006 μM compared to 0.204 ± 0.011 μM). Notably, NMDA (30 μM) applied in the superfusate for 12 min (during stimulations S5 to S8) had no effect on the stimulated release. One-way ANOVA showed no change in magnitude of stimulation across the experiment ($F(2.82,19.74) = 1.070$; $p = 0.3811$: Greenhouse-Geisser corrected: Figure S1a). Moreover 1-way ANOVA of the summary data showed no significant differences between baseline (stimulations S1 to S4), Drug (S6 to S8) and Washout (S12 to S14) (F(2,21 = 0.839; $p = 0.446$: Figure S1b), nor of peak responses during Baseline (stimulus S4), Drug (stimulus S8) and Washout (stimulus S14)($F(2,21) = 2.449$; $p = 0.111$: Figure S1c).

Attenuation of stimulated accumbal dopamine release by NMDA is mediated through metabotropic glutamate receptors

Felicity S.E. Spencer, Maria Glodkowska, Anna I Sebold, Ersin Yavas, Andrew M.J. Young

Conclusion: Therefore, NMDA has no effect on electrically-stimulated dopamine release during low frequency stimulation, contrasting with the attenuation of the effect seen during high-frequency stimulation.

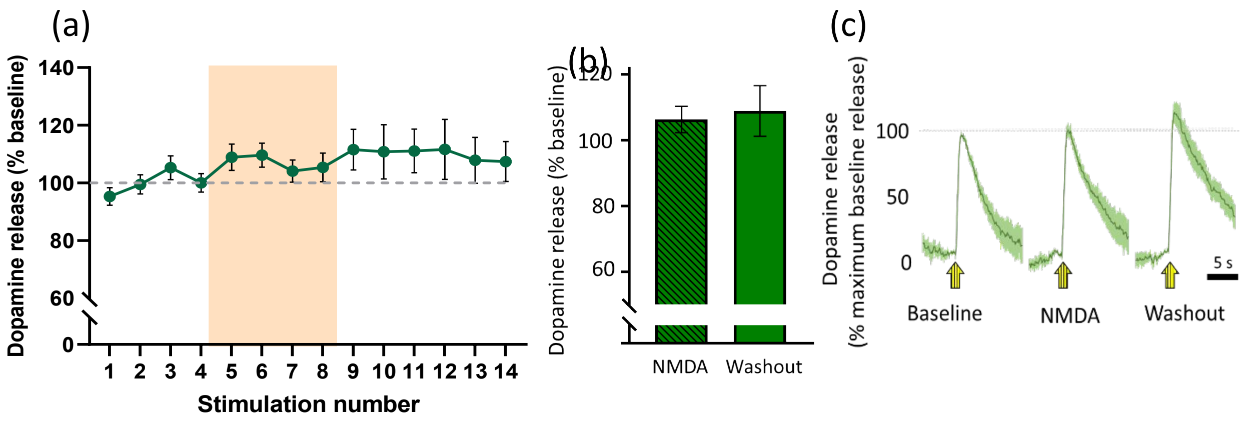

Figure S1 : Effect of NMDA (30 µM) on electrically stimulated dopamine release, using low frequency stimulation. *(a) Time course of stimulated release, over repeated electrical stimulations (5 x 1 ms pulses; 10 Hz, 800 µA) at 3 min intervals. NMDA (30 µM: n = 8) was applied for 12 min, during stimulations S5 to S8 (shaded panel). (b) Summary data showing mean ± SEM responses over stimulations 1 to 4 (Baseline), 6 to 8 (NMDA) and 12 to 14 (Washout). (c) Pooled (mean ± SEM) current vs time plots for the 15 sec recording period during stimulation S4 (Baseline), S8 (NMDA) and S14 Washout). All data are normalised to the maximum current response during baseline stimulation for each slice. Stimulation application is indicated by the yellow arrow. No statistically significant differences were found.*
